# How Bacterial Communities Expand Functional Repertoires

**DOI:** 10.1371/journal.pbio.0040430

**Published:** 2006-12-12

**Authors:** James Versalovic, David Relman

## Abstract

Probiotics, live microorganisms administered to improve health, interact in as yet poorly understood ways with the complex microbial communities that inhabit the human host.

Complex microbial communities, such as biofilms in the oral cavity and lumenal and mucosal communities in the gastrointestinal tract, play prominent roles in human health and disease [1]. Microbial communities in vivo include many different bacterial species that are in dynamic, intimate association with each other and with the human host. In humans, the intestinal microbiota is composed of well over 500 species [2], and the concept of humans as super-organisms [1,3] is highlighted by estimates that the human microbiome contains roughly 100 times as many genes as does the human genome. Increasingly, live microorganisms—probiotics—are being administered in order to promote human health. But much remains to be understood about the nature of the molecular interactions between newly arrived and resident microbial community members. Can microbial communities be effectively manipulated by administering defined dosages of a specific probiotic? How do probiotics affect the functional properties of indigenous microbial communities?

## The Intestinal Microbiota

The microbiota of animals and humans begins to be established as soon as the newborn is exposed to the environment at birth; its complete establishment in the mammalian intestine requires months. Pioneering bacteria that appear in the mammalian intestine within the first several days of life are followed by a succession of colonizing species in a process that continues at least through weaning [4,5]. Diet, host genotype, social group, medical history, and advanced age have all been shown to influence the structure of the human intestinal microbiota [6]. In general terms, phylogenetic analysis of mammalian microbial communities especially in the intestine reveals shallow, fan-like radiations, revealing only a handful of phyla (Firmicutes, Bacteroidetes, Actinobacteria, and Proteobacteria are most prominent). Most of the taxonomic diversity is found at the level of species and strains, with a highly uneven distribution of abundance among taxa [1]. It is estimated that over 80% of the species in the human colon are not yet cultivated [2].

New molecular tools are facilitating a more detailed view of microvariation in structure and function that may be important both for the microbial community and for metazoan physiology [5,7]. Differences between the colonic mucosa–associated and fecal microbiota of a single individual, as well as larger degrees of variation at a single site between individuals, suggest that compartmentalization and biogeography, as well as host-related factors, influence the microbial community at any given site [2,8].

Given the complexity of both the communities themselves and the range of factors known to influence them, experiments with model systems have made important contributions to our understanding of host–microbe interactions. In particular, the use of conventionally reared, microbe-free, and gnotobiotic mice have allowed controlled experiments that would be impossible in humans. Many of our current insights into the workings of probiotics come from mouse studies. Mouse models of colitis have described important compositional and functional differences between intestinal Lactobacillus populations. Lactobacillus spp. have featured prominently as probiotics. Interleukin-10–deficient mice predisposed to colitis contained predominantly Lactobacillus johnsonii, whereas mice with an intact interleukin-10 gene lacking evidence of colitis had greater Lactobacillus species diversity and featured L. reuteri as a predominant species [9,10]. Lactobacillus species differences were correlated to functional differences in the abilities of these commensal bacteria to suppress host cytokine production. Intestinal L. reuteri isolates from healthy mice were able to suppress tumor necrosis factor-alpha (TNF-&alpha;) production by macrophages, whereas L. johnsonii isolates from diseased animals were not able to affect microphage TNF-&alpha; production[11].

The human microbiome contains roughly 100 times as many genes as does the human genome.

## Altering the Mix

The administration of a single bacterial species in large doses to an animal with an established, complex, indigenous intestinal microbiota with hundreds of species does not necessarily lead to successful colonization. Studies in humans have showed that probiotic strains generally persist in the human intestine for only a short time beyond when they are actually being ingested [12]. However, when colonization is achieved, the result may be communal population shifts with important consequences for gastrointestinal physiology. Pathogens that have adapted to mucosal surfaces may be particularly adept at competing with the resident community. In one study, oral administration of the murine pathogen Helicobacter hepaticus to mice resulted in reproducible, long-lasting shifts in intestinal microbial composition and reduced overall diversity of the intestinal microbiota [13]. The changes reflect in part the ability of the exogenously administered enteric pathogen to attain numerical dominance in the community. Although global metabolic functions of the community were not measured in this study, it is likely that large-scale changes in the microbial community's functional repertoire occurred. Changes in the composition of microbial communities can result in aggregate functional differences that may affect digestion and host mucosal biology.

In general, there are both direct and indirect mechanisms by which the introduction of one organism might favorably affect a second organism. The first organism might provide a nutrient or other product (such as an enzyme) that can be used or recognized by the second, resulting in changes in gene expression and metabolism. In addition, the first might provide binding sites or other factors necessary for colonization by the second. Less-direct mechanisms might involve effects by the first organism, mediated by the community (e.g., suppressing a competitor) or by the host (e.g., altering immune system activity), resulting in more favorable environmental conditions for the second organism.

Recent studies are shedding light on the important role of the intestinal microbiota in modulating the impact of exogenously administered bacteria. The bacterium Bacillus thuringiensis produces a potent insecticidal toxin and has been used to manage insect populations that interfere with the practice of forestry and agriculture. After it is ingested by an insect, B. thuringiensis requires the presence of commensal midgut bacteria to produce insecticidal activity, as demonstrated by the loss of activity following pretreatment of moth larvae with antibiotics [14]. Functional activities that have been long assumed to reside within one microbe may in fact result from the interactions of many different microbes within a given habitat.

The deliberate manipulation of microbial communities may result in sustainable changes of aggregate microbial and host physiologic functions. The administration of the probiotic L. paracasei to Trichinella spiralis–infected mice restored profiles of host energy metabolism, fat mobilization, and amino acid metabolism to those seen in mice without T. spiralis infection [15]. The administration of prebiotics may also result in alterations to host physiology through changes in microbial composition. Prebiotics are nonliving food ingredients that selectively promote the growth of certain beneficial members of the indigenous intestinal microbiota and are typically not digestible by the host, per se. The introduction of specific prebiotics such as inulin, oligofructose, galacto-oligosaccharides, and lactulose clearly alter the microbial composition of the mammalian large intestine [16]. Some of the most commonly used prebiotic carbohydrates are fermented by colonic microbes to produce short-chain fatty acids, which are known to affect microbial and host mucosal physiology. Some prebiotics are believed to cause large shifts in microbial populations by facilitating the selective proliferation of bifidobacteria and lactobacilli. Changes in the host such as increased calcium absorption and immunomodulation may be partly or entirely secondary to alterations in microbial composition.

## Functional Genomic Approaches

The combined functional genomic repertoire of entire microbial communities (i.e., the microbiome) can now be accessed with metagenomic approaches, providing new opportunities for scientists to explore mammalian physiology in a host-wide integrated manner. Using such a metagenomic approach, Gill et al. recently found that the human distal gut microbiome is enriched for many genes contributing to glycan, amino acid, and xenobiotic metabolism, and that this microbial genetic assemblage provides Homo sapiens with a vast set of genes that effectively increases the human physiological repertoire [3].

The manuscript in this issue of *PLoS Biology* by Sonnenburg et al. [17] examines the functional interactions between an intestinal mutualist, Bacteroides thetaiotaomicron, and an organism commonly used as a probiotic, Bifidobacterium longum, in a mouse model. Changes in function are examined at a global level by using a variety of genomic approaches both to monitor transcriptional changes and to characterize habitat-associated carbohydrates. Ba. thetaiotaomicron appears able to target a greater diversity of polysaccharides for degradation when Bi. longum is present. When present, the probiotic also induces an array of host innate immunity genes. Prior work by the same group described functional genomic changes in commensal bacteria that enabled them to use host glycans when dietary carbohydrates were scarce [18]. Intestinal commensals are capable of altering their own genetic program depending on environmental variables, but can commensal and probiotic bacteria alter each other's genetic programs in the milieu of the mammalian intestine? The answer appears to be yes.

Technical approaches for understanding the composition and functions of the intestinal microbiota are being expanded to meet the challenges of many tall tasks ahead. The rapid completion of many microbial genomes provides ample DNA sequence information for functional metagenomics. Additional metagenomic studies of intact microbial communities are generating genetic information that is not found in completed or draft single-organism genome projects. The “mapping” of gene sequences and their predicted products to “clusters of orthologous groups” or on known metabolic pathways (e.g., using the KEGG database) allows predictions to be made regarding the functional capabilities of a microbial community [3]. As the era of high-throughput sequencing-by-extension unfolds, DNA sequence information that is pertinent to mammalian microbiomes will rapidly proliferate. Functional metagenomics will benefit from DNA microarrays that include amalgamations of genes from multiple strains of a single species, from the multiple species that comprise microbial communities, and from genes from the host. Work of this sort will highlight pathways affected by deliberate manipulations with probiotics. Advances in bioinformatics will facilitate correlation of community-wide gene expression profiling with pathways or system modules. Strategies for cataloging microbial communities (such as denaturing high-performance liquid chromatography and high-throughput rDNA clone library sequence analysis) enable investigators to create profiles of the intestinal microbiota and to assess its dynamic features secondary to environmental influences. Fluorescence in situ hybridization applications are increasing our appreciation for the spatial topography of bacteria within intestinal communities and provide new insights into nonrandom distributions of organisms and interspecies interactions in space. Cell sorting and microfluidics-based microdevices enable microbiologists to sort and isolate individual bacterial cells and to study the biological properties of organisms directly from natural communities, including microbial biofilms. The ability to acquire a genome sequence and genome-wide transcript abundance patterns from a single bacterial cell, without the need for cultivation in the laboratory, will have a huge impact on our ability to understand the role of cultivation-resistant organisms in their natural community setting. Finally, metabolomics and proteomics will facilitate explorations of secreted compounds and polypeptides that may serve as intermicrobial and microbial-host signals.

Fundamental questions are being explored regarding the functional capabilities of microbial communities that directly affect the physiology of the mammalian intestine and the entire host. Sustainable effects on the host may depend on lasting changes in microbial community composition, its metagenome, and, more specifically, on alterations in the metabolic profile of the community. As we improve our understanding of how probiotics might alter the functions of the resident microbiota, clinicians may improve their abilities to select candidates (microbial and host) for probiotic therapy, with the goal of enhancing or restoring the health of patients.
